# Outcomes after stereotactic radiosurgery of brain metastases in patients with malignant melanoma and validation of the melanoma molGPA

**DOI:** 10.1007/s12094-021-02607-8

**Published:** 2021-05-15

**Authors:** K. A. Kessel, A. Deichl, J. Gempt, B. Meyer, C. Posch, C. Diehl, C. Zimmer, S. E. Combs

**Affiliations:** 1 Department of Radiation Oncology, Klinikum rechts der Isar, Technical University Munich (TUM), Ismaninger Straße 22, 81675 Munich, Germany; 2 Institute of Radiation Medicine (IRM), Helmholtz Zentrum München, Neuherberg, Germany; 3 Deutsches Konsortium für Translationale Krebsforschung (DKTK), DKTK Partner Site Munich, Munich, Germany; 4 Department of Neurosurgery, Technical University of Munich (TUM), Munich, Germany; 5 Department of Dermatology and Allergy, Technical University of Munich (TUM), Munich, Germany; 6 Department of Neuroradiology, Technical University of Munich (TUM), Munich, Germany; 7 Faculty of Medicine, Sigmund Freud University, Vienna, Austria

**Keywords:** Brain metastases, Melanoma, SRS, Radiosurgery, GPA, Prognostic factors

## Abstract

**Introduction:**

Malignant melanoma is the third most common primary in the diagnosis of brain metastases. Stereotactic radiosurgery (SRS) is a well-established treatment option in limited brain disease. We analyzed outcomes of SRS with a particular focus on the graded prognostic assessment (GPA, melanoma molGPA), prognostic factors, and toxicity.

**Methods:**

We evaluated 173 brain metastases in 83 patients with malignant melanoma. All were treated with SRS median dose of 20 Gy prescribed to the 80 or 100% isodose line between 2002 and 2019. All patients were followed-up regularly, including contrast‐enhanced brain imaging as well as clinical examination, initially 6 weeks after treatment, then in quarterly follow-up.

**Results:**

The median age was 61 years (range 27–80); 36 female and 47 male patients were treated. After a median follow-up of 5.7 months, median OS (overall survival) was 9.7 months 95%-KI 4.7–14.7). LC (local control) at 6 months, 12, 24 months was 89%, 86%, and 72%, respectively (median was not reached). Median DBC (distant brain control) was 8.2 months (95%-KI 4.7–11.7). For OS, a KPS ≥ 80%, a positive BRAF mutation status, a small PTV (planning target volume), the absence of extracranial metastases, as well as a GPA and melanoma molGPA > 2 were prognostic factors. In the MVA, a small PTV and a melanoma molGPA > 2 remained significant.

**Conclusion:**

The present survival outcomes support the use of the disease-specific melanoma molGPA as reliable prognostic score. Favorable outcomes for SRS compared to other studies were observed. In the treatment of brain metastases of malignant melanoma patients, a multidisciplinary approach consisting of surgery, SRS, chemotherapy, and immunotherapy should be considered.

## Introduction

Patients with solid malignancies often develop brain metastasis at some point during their disease. Metastases are the most common brain neoplasm and may spread from any primary disease [1]. In up to 40% of patients with solid primary tumors outside the central nervous system, brain metastases are diagnosed, and the incidence is continuously increasing [2]. Only 10% of patients survive more than 1 year [3].

The treatment of brain metastases relies on local strategies, including surgery, stereotactic radiosurgery (SRS), as well as on whole-brain radiation (WBRT). First-line treatment decisions in patients with newly diagnosed brain metastases mainly rely on the primary tumor, number and size of brain metastases and Karnofsky performance score (KPS), which are considered in the newly established graded prognostic assessment (GPA). Primarily the GPA was developed as a new score in 2008 and based on an analysis of 1960 patients whose data were extracted from the Radiation Therapy Oncology Group (RTOG) database. The GPA includes four criteria: age, KPS, number of brain metastases, and the presence/absence of extracranial metastases [4]. Based on a retrospective study of > 4000 patients with brain metastases, Sperduto et al. determined various prognostic factors depending on the histology of the primary and modified the GPA to so-called disease-specific GPA (dsGPA) scores. For patients with malignant melanoma, the updated prognostic score is a further development of the GPA—the so-called melanoma molGPA—is a more complex assessment, which also includes the BRAF mutation status [5–7].

SRS has become increasingly important, and a well-accepted treatment method for brain metastases [8, 9]. The technique relies on multiple beams of radiation intersecting at a target precisely located within three dimensions [8]. For brain metastases, SRS can be delivered via linear accelerator or gamma knife [10] and is now used in a variety of clinical scenarios for patients with brain metastases, including as a boost WBRT therapy, as a definitive treatment alone for patients with a limited number of brain metastases, and in the pre- or postoperative setting [10].

Malignant melanoma is the third most common primary in the diagnosis of brain metastases after lung carcinoma, and breast cancer [11]. Brain metastases occur in up to 50% of patients with metastatic melanoma [12]. The chances of being cured are good in the early stages of the disease, but the median overall survival (OS) of patients with untreated brain metastases is less than 3 months [13].

Up until 2011, no systemic therapy had convincingly been shown to improve the survival of patients with metastatic melanoma [14]. Since the last decade, two different treatment concepts are available for targeted therapy, BRAF ± MEK inhibition [15, 16], and immune checkpoint blockade [17, 18], which confirmed a survival benefit for advanced melanoma patients [14]. They represent two very distinctive classes of agents: small molecule inhibitors of the MAP kinase signaling pathway and monoclonal antibodies against immune checkpoint molecules [14]. In 40–60% of malignant melanomas, there is a mutation in the BRAF gene that leads to the activation of a signal transduction pathway relevant for tumor development [19]. Since the drugs impressively control extracranial metastasis, the therapy of brain metastasis is of new relevance [20]. Additionally, the availability of further novel immunotherapeutic drugs will be upcoming, which will continuously improve outcomes. Therefore, the need for non-invasive, highly effective treatments for brain metastases becomes more imminent.

Malignant melanoma brain metastases have traditionally been considered ‘radioresistant’ to conventional fractionated external beam radiotherapy (FSRT) and WBRT [21]. Hence, in the past decade, SRS has become a well-established treatment modality with high efficacy as it delivers a high dose to the lesion [21]. In selected patients with one to four metastases measuring less than 3–4 cm, SRS yields an excellent local and distant brain control (LC, DBC), more than 85%, and median survival of 5–11 months [22].

A multidisciplinary approach consisting of surgery, radiotherapy, chemotherapy, and immunotherapy has proved beneficial in advanced stages of metastasis of malignant melanoma [23].

In the present study, we analyze outcomes (OS, LC, DBC) after SRS of brain metastases in patients with malignant melanoma. We further aim to investigate the graded prognostic assessment (GPA, melanoma molGPA), prognostic factors, and toxicity.

## Patients and methods

### Patients

We retrospectively evaluated 173 brain metastases and 83 patients with malignant melanomas. All were treated with SRS at the Department of Radiation Oncology, Technical University of Munich, between 2002 and 2019. Table 1 displays patient characteristics. The ethics committee of the Medical Faculty of the Technical University Munich (TUM) approved this study and waived patient informed consent: 257/16 S (01.06.2016).

**Table 1:** Patient characteristic (patients *n* = 83, lesions *n* = 173)

Description	Value	%
Age at RT [years]		
Median	61	
Range	27–80	
Gender		
M	47	56.6
F	36	43.4
BRAF mutation		
Yes	19	22.9
No	27	32.5
Unknown	37	44.6
KPS at RT [%]		
100	17	9.8
90	77	44.5
80	52	30.1
70	23	13.4
60	3	1.7
50	1	0.6
Simultaneously immune therapy		
Yes	11	13.3
No	72	86.7
Immune and BRAF therapy agent		
Ipilimumab	1	9.1
Nivolumab	3	27.3
Nivolumab & Ipilimumab	3	27.3
Pembrolizumab	2	18.2
Vemurafenib	2	18.2
Parallel chemotherapy		
Yes	2	2.4
No	81	97.6
Chemotherapy agent		
Temozolomid	1	50.0
Fotemustin	1	50.0
Prior WBRT		
Yes	17	20.5
Dose [Gy]	30/10	70.6
Dose [Gy]	40/20	29.4
No	66	79.5
Number of metastases at RT		
1	29	16.8
2–3	78	45.1
≥ 4	66	38.2
Extracranial metastases		
Yes	66	79.5
No	17	20.5
GPA		
0–1.0	43	24.9
1.5–2.0	99	57.2
2.5–3.0	27	15.6
3.5–4.0	4	2.3
Melanoma molGPA		
0–1.0	26	15.0
1.5–2.0	101	58.4
2.5–3.0	36	20.8
3.5–4.0	10	5.8
Time from primary diagnosis to brain metastasis [months]		
Median	36.3	
IQR	81.8	
PTV [ml]		
Median	1.0	
IQR	2.9	
D_max_ (PTV) [Gy]		
Median	24.9	
IQR	1.7	

° Number according to patients, *KPS* Karnofsky performance score, *BRAF*  Serine/threonine protein kinase, encoded on chromosome 7q34, that activates the MAP kinase/ERK-signaling pathway, *GPA* Graded prognostic assessment according to [24, 25], *Melanoma molGPA*  Disease specific GPA for melanoma patients according to [26], *WBRT* Whole brain radiotherapy, *PTV* Planning target volume, *IQR* Interquartile range

### Treatment

SRS treatment was performed using a thermoplastic mask system (Brainlab, Germany) and daily image-guidance (IGRT) by robotic ExacTrac positioning (Brainlab, Germany). Within the stereotactic localization system, contrast-enhanced computed tomography (CT) and T1-weighted magnetic resonance images (MRI) were acquired with a slice thickness of 3 mm.

We fused the images and defined the planning target volume (PTV) as residual macroscopic tumor tissue with a margin of 1–2 mm for residual uncertainties.

We applied a median total dose of 20 Gy (range: 18–20 Gy) prescribed to the 80 or 100% isodose line. A Clinac Trilogy linear accelerator equipped with a 120 HD multi-leaf collimator (Varian Medical Systems, Palo Alto, CA, USA) and 6 MV photons was used for irradiation. Eleven patients (13.3%) received a simultaneously immune therapy or BRAF inhibitor. Parallel chemotherapy was administered in two patients (2.4%). Before SRS, 17 (17/83, 20.5%) patients were already pre-treated with WBRT within a median time interval of 98 days (range 15 days–3.2 years) prior to SRS and a median total dose of 30 Gy (range: 30–45 Gy).

### Follow-up

All patients were followed-up regularly, including contrast‐enhanced brain MRI (T1-weighted) and a clinical examination. The first follow-up was performed 6 weeks after the end of treatment, then in a quarterly follow-up with physical examination, blood chemistry and magnetic resonance imaging for local control. After 2 years of recurrence‐free survival, the intervals were prolonged individually. In the case of local or distant intracranial failure, salvage therapy was performed after interdisciplinary discussion (neurosurgical intervention with/without adjuvant radiation therapy performed as WBRT or radiosurgery). Leptomeningeal failure and radiation-induced brain necrosis were confirmed by an interdisciplinary board and determined either after surgery and histopathological examination or on MRI.

Neurologic toxicities were classified as acute if they occurred during treatment or up to the first 6 weeks after the end of SRS. If they occurred later, they were considered as late toxicities.

### Statistics

Primary endpoints were OS, LC (defined as the time to first local failure (LF), which is determined by new contrast enhancement of the previously irradiated metastasis on MRI confirmed by experienced radiologists), and DBC (defined as the time to first loco-regional, intra-cranial failure (LRF), hence, the growth of new or not-treated brain metastases). Secondary objectives included the assessment of GPA (initial GPA and melanoma molGPA, Table 3) and the analysis of prognostic factors as well as neurologic toxicity.

Continuous data were expressed as median and IQR (interquartile range), categorical data as frequency counts and percentages. For calculation of outcomes, we used all treatment cases for LC (*n* = 173) and the first treatment for OS and DBC (*n* = 83). We calculated OS from the last treatment day until the last follow-up visit or death; LC/DBC was calculated from the last treatment day until the date of local/distant brain progression, last follow-up visit, or death. All statistical calculations were performed using SPSS Statistics Version 25 (IBM, USA). Outcome analyses and determination of prognostic factors were based on Kaplan–Meier estimates with log-rank tests and the Cox regression method.

A *p* value of < 0.05 was defined as the threshold for statistical significance within a confidence interval of 95%.

## Results

### Outcomes and toxicity

The median age was 61 years (range 27–80); 36 females (36/83, 43.4%) and 47 (47/83, 56.6%) male patients were treated with SRS. Median follow-up for all patients was 5.7 months (IQR: 14.5 months); for patients alive, it was 24.6 months (IQR: 25.1 months). Seven patients (7/83, 8.4%) were lost to follow-up.

At the time of analysis, 74.7% of patients (62/83) were deceased, which resulted in a median OS of 9.7 months (95%-KI 4.7–14.7). Twenty-two patients (22/173, 12.7%) suffered from local failure. LC at 6-months, 12-, and 24-months was 89%, 86%, and 72%, respectively (the median was not reached). Loco-regional failure occurred in 41 patients (41/83, 49.4%), resulting in a median DBC of 8.2 months (95%-KI 4.7–11.7). Table 2 shows further outcome data.

**Table 2:** Median and life table for OS, LC, and DBC

	Median [months]	The proportion of patients surviving after			
		6 months	12 months	18 months	24 months
OS	9.7	67%	46%	35%	32%
LC	–	89%	86%	82%	72%
DBC	8.2	56%	44%	34%	26%

Radionecrosis was seen by MRI in 9 (9/83, 10.8%) patients after SRS classified as CTCAE grade 2 in eight cases as CTCAE grade 1 in one case. The median time to occurrence of radionecrosis was 9.0 months (range 2.4–18.8 months). Leptomeningeal failure did not occur in this cohort. Regarding side effects CTCAE grade ≥ 3, we saw motor disorders in four cases in the first 6 months, and in three after 6 months. No sensory disorders grade ≥ 3 were reported. Epilepsy was seen in one patient in the first 6 months and in two patients after six months. Other side effects grade ≥ 3 were nausea, cognitive disorders, and dizziness in one patient each.

### GPA assessment

We calculated the initial GPA and melanoma molGPA, according to the recently published works by Sperduto et al. [4]. The GPA is calculated by adding the score values for the three parameters for age, KPS, extracranial metastases, and the number of brain metastases; the melanoma molGPA by also adding the value of the BRAF status (Table 3). Each of the four criteria is given a score of 0, 0.5, or 1.0, with the best prognosis in patients with a score of 4.0.

**Table 3:** Calculation scheme for the GPA and melanoma molGPA

	Prognostic factor		Score value
GPA	Age [years]	< 50	1
		≥ 50 < 60	0.5
		≥ 60	0
	KPS [%]	100–90	1
		80–70	0.5
		≤ 60	0
	Extracranial metastases	No	1
		Yes	0
	Number of brain metastases	1	1
		2–3	0.5
		≥ 4	0
Melanoma molGPA	Age [years]	< 70	0.5
		≥ 70	0
	KPS [%]	100–90	1
		80	0.5
		≤ 70	0
	Extracranial metastases	No	1
		Yes	0
	Number of brain metastases	1	1
		2–4	0.5
		≥ 5	0
	BRAF mutation status	Positive	0.5
		Negative/unknown	0

### Prognostic assessment

**Fig. 1:**
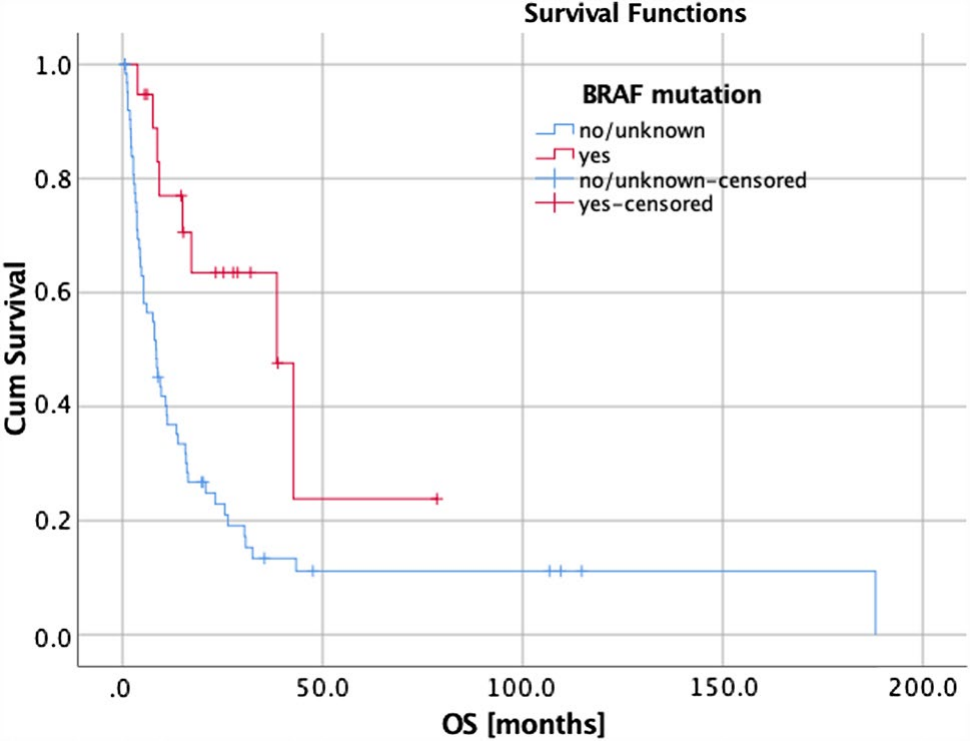
Survival curves for BRAF mutation status (*p* = 0.004)

**Fig. 2:**
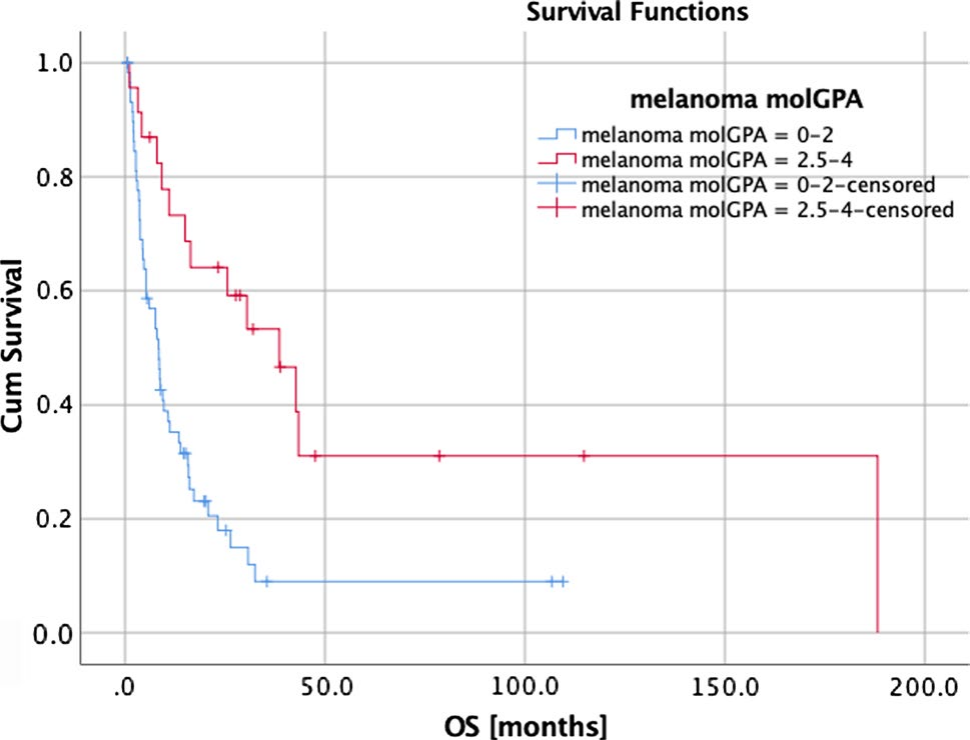
Survival curves for the melanoma molGPA (*p* = 0.001)

**Fig. 3:**
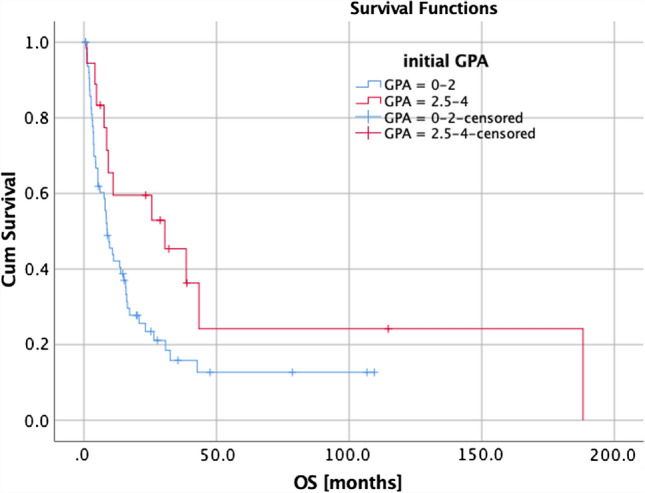
Survival curves for the initial GPA (*p* = 0.043)

Univariate and multivariate analyses (UVA, MVA) are shown in Table 4. For OS, a KPS ≥ 80%, a positive BRAF mutation status (Fig. 1), a small PTV, the absence of extracranial metastases, as well as a GPA and melanoma molGPA > 2 (Fig. 2) were prognostic factors. In the MVA, we used only the significant values and excluded two parameters: the initial GPA and its values should only be included once in the MVA, and the extracranial metastases, BRAF status, and KPS as they are part of the molGPA calculation. In the MVA, a small PTV and a melanoma molGPA > 2 remained significant (Fig. 3).

**Table 4:** Prognostic assessment on OS, LC, and LRC

	OS				LC		DBC	
	UVA		MVA		UVA		UVA	
	*P*-value	HR (95% CI)	*P*-value	HR (95% CI)	*P*-value	HR (95% CI)	*P*-value	HR (95% CI)
Age at RT*	0.707	0.99 (0.98–1.02)	–		0.201	0.98 (0.94–1.01)	0.642	0.99 (0.97–1.02)
KPS (< 80% vs. ≥ 80%)	0.024*	2.28 (1.11–4.65)	–		0.299	0.04 (0.00–16.77)	0.680	0.74 (0.18–3.09)
Time from primary diagnosis to brain metastasis *	0.186	1.00 (0.99–1.00)	–		0.562	1.00 (0.99–1.00)	0.770	1.00 (1.00–1.01)
BRAF mutation (yes vs. no/unknown)	0.004*	3.04 (1.44–6.40)	–		0.703	1.18 (0.50–2.80)	0.834	0.93 (0.49–1.78)
BRAF mutation (yes vs. no)	0.050	0.44 (0.19–1.00)	–		0.316	0.51 (0.14–1.90)	0.929	0.97 (0.46–2.03)
simultaneously immune therapy (yes vs. no)	0.478	1.14 (0.79–1.66)	–		0.624	1.15 (0.67–1.97)	0.862	0.97 (0.65–1.43)
Prior WBRT (yes vs. no)	0.061	0.76 (0.56–1.01)	–		0.086	0.67 (0.43–1.06)	0.982	1.00 (0.64–1.54)
PTV*	< 0.001*	1.12 (1.05–1.19)	0.001*	1.11 (1.04–1.18)	0.045*	1.09 (1.00–1.19)	0.394	1.05 (0.94–1.16)
D_max_*	0.163	0.86 (0.70–1.06)	–		0.027*	0.67 (0.47–0.96)	0.058	1.34 (0.99–1.81)
Extra-cranial metastases (yes vs. no)	0.004*	0.57 (0.39–0.84)	–		0.065	0.50 (0.24–1.04)	0.053	0.67 (0.44–1.01)
Number of all brain mets (1 vs. > 1)	0.538	0.83 (0.46–1.50)	–		0.164	0.51 (0.20–1.32)	0.037*	2.52 (1.06–6.01)
GPA (0–2 vs. 2.5–4)	0.043*	1.97 (1.02–3.81)	–		0.736	0.84 (0.31–2.29)	0.044*	0.43 (0.19–0.98)
Melanoma molGPA (0–2 vs. 2.5–4)	0.001*	0.34 (0.18–0.63)	0.001*	2.92 (1.54–5.54)	0.996	1.00 (0.43–2.38)	0.409	1.32 (0.68–2.56)

*KPS* Karnofsky performance score, *BRAF*  Serine/threonine protein kinase, encoded on chromosome 7q34, that activates the MAP kinase/ERK-signaling pathway, *GPA*  Graded prognostic assessment according to [27], *WBRT* Whole brain radiotherapy, *PTV* Planning target volume, *D*_*max*_ Maximum dose to the PTV, *UVA* Univariate analysis, *MVA* Multivariate analysis, * continues variable

A small PTV and *D*_max_ had a significant influence on LC. The presence of a single brain metastasis, and the initial GPA impacted DBC.

## Discussion

The present study aimed to identify outcomes and prognostic factors of melanoma patients with brain metastasis. Treatment was very well tolerated with low rates of side effects, especially regarding high-grade toxicity. Median OS was 9.7 months, and LC at one year was 86%. The melanoma molGPA showed a significant impact on OS with *p* = 0.001 with a higher molGPA associated with an improved OS. Further predictors of outcome include a KPS ≥ 80%, a positive BRAF mutation status, a small PTV, the absence of extracranial metastases.

Predictors for OS in patients with malignant melanoma metastatic to the brain have been published previously and include LDH levels, age, KPS, number of brain metastasis, leptomeningeal spread, presence of extracranial metastases, melanoma ulceration, histology and neurologic symptoms [28]. Median OS in our study was 9.7 months, which is equal compared to the study of Yu et al. in 2002 [4, 28] as well as Raizer et al. in 2008 [4] and close to other relevant studies in this field (Table 5). In our study, LC, after one year, was 86%, it should be mentioned that early death may preclude the development of progression. Other groups, Gaudy-Marqueste et al. and Mori et al., achieved comparable excellent control rates of 84% and 90% [29–34]. Powell et al. reported an LC rate of 63% in 50 examined patients [35]. This OS and LC rate reinforces the statement that SRS has become a well-established treatment modality with high efficacy as it delivers a high dose to the lesion, even if brain metastases of malignant melanoma have traditionally been considered radioresistant lesions when treated with conventional radiotherapeutic modalities [32].

**Table 5:** Selection of studies with local control rates after 1 year

Publication, Year	Patients	Primary	Therapy	Median OS [months]	LC after 1 year
Yu et al. [35], 2002	122	MM	GKR	9.1	86% (< 1 cm), 63% (3–9 cm)
Gaudy-Marqueste et al. [37], 2006	106	MM	GKR	4.1	69%
Neal et al. [59], 2014	129	MM	GKR	NR	81%
Rades et al. [60], 2014	54	MM	SRS	NR	100% (21–22.5 Gy), 72% (20 Gy)
Gieger et al. [61], 1997	12	MM	SRS	NR	57%
Brown et al. [62], 2008	39	MM/RCC	SRS	14.2	100% (6 months)
Mori et al. [38], 1998	60	MM	SRS	7.0	90%
Radbill et al. [63], 2004	51	MM	GKR	5.7	81%
Lavine et al. [64], 1999	45	MM	GKR	43.0	97%
Sia et al. [65], 2015	162	various	SRS	5.1	82%
Likhacheva et al. [66], 2013	251	various	SRS	11.1	95%
Chang et al. [67], 2009	58	various	SRS	15.2	67%
Andrews et al. [42], 2004	331	various	WBRT + SRS Boost	NR	82%
This work	83	MM	SRS	9.7	86%

*MM* Malignant melanoma, *RCC*  Renal cell cancer; *SRS* Stereotactic radiosurgery, *GKR* Gamma knife radiosurgery, *NR* Not reported

None of the tested factors impacted LRC, including WBRT. However, this offers only limited information for the present cohort as only 21% of patients were pre-treated with WBRT.

This has to be seen in contrast to other studies of different tumor types which have demonstrated increased distant brain control with WBRT. Presumably this is a dose effect with malignant melanoma and thus shows similar results to the paper published in 2019 by Hong et al. that adjuvant WBRT does not provide clinical benefit in terms of distant intracranial control [36].

Regarding radionecrosis, it occurred in 10.8% of our patients after SRS, which is comparable with other studies [37, 38]; all cases of low CTCAE grade.

The results showed that OS was significantly impacted by the BRAF mutation status (*p* = 0.004), with a positive BRAF mutation status associated with an improved OS. However, it offers only limited information for the present cohort as 45% of patients had an unknown BRAF mutation status.

Bian et al. evaluated in 2016, 401 patients with metastatic melanoma treated with SRS for metastases to the brain. A higher tumor volume was associated with worse survival, as in our study [39]. In multivariate analysis, a small PTV and a melanoma molGPA > 2 remained significant. Historically, the original GPA has been calculated with the four parameters: age, KPS, number of brain metastases, and extracranial metastases (Table 3). It has been proven useful by Sperduto et al. in a study with 140 patients [21]. This impact could also be shown in our present evaluation (*p* = 0.043). After further research, Sperduto et al. identified five significant prognostic factors for survival (age, KPS, extracranial metastases, number of brain metastases, and BRAF status) for melanoma patients, and rebuilt the initial GPA to the melanoma molGPA [40–45]. They validated the score on a cohort of 823 melanoma patients with newly diagnosed brain metastases in a multi-institutional retrospective analysis. This review demonstrates that this disease-specific assessment, including relevant outcome and prognostic biomarkers, provides a better and reliable prognostic score. This strengthens the trend that incorporating biomarkers, which show influence on OS based on the latest research, will facilitate clinical decision making regarding whether and which treatment is appropriate. It will also be useful for stratification of future clinical trials [46].

Immunomodulators and targeted agents against mutations in the BRAF gene have become established treatment for patients with metastatic melanoma, offering a survival benefit [4]. The substances also appear to have a positive effect on intracerebral control. Dummer et al. 2014 in their small collective (*n* = 24) treated with vemurafenib, 37% achieved an intracranial tumor decrease of > 30% [47]. In the review of Ly et al. [28], treatment with BRAF inhibitors was associated with improved local control after SRS in 52 patients with melanoma and brain metastases compared to no treatment of BRAF inhibitors.

Further prognostic factors such as the number and symptoms of cerebral metastases, serum LDH and S100 levels, extracranial metastasis, and KPS receive more and more attention in interdisciplinary therapy planning. The trend is moving more and more in the direction of individualized treatment. Personalized medicine has the potential to tailor therapy with the best response and highest safety margin to ensure better patient care. By enabling each patient to receive earlier diagnoses, risk assessments, and optimal treatments, Personalized medicine holds promise for improving health care while also lowering costs [14]. Current clinical studies are investigating new therapy options for melanoma patients with brain metastases such as PD-1 antibodies, ipilimumab plus nivolumab, BRAF inhibitors plus MEK inhibitors, as well as stereotactic radiotherapy in combination with immunotherapy or targeted therapy [48]. Ipilimumab, pembrolizumab, and nivolumab are approved for the treatment of unresectable or metastatic melanoma, as is the combination of nivolumab and ipilimumab, which in the CheckMate 067 trial outperformed the respective monotherapies in terms of response and progression-free survival, even though it was significantly more toxic [49]. Therapy with an antibody directed against PD-1 with or without the addition of ipilimumab is the standard therapy at least for advanced melanoma without BRAF mutation. The combination of BRAF/MEK inhibitors shows the highest response rates of all melanoma therapies. This treatment is particularly suitable as first-line therapy for patients with symptomatic BRAF mutated melanoma with rapid progression and high therapeutic pressure. However, the duration of therapy is usually limited by the development of resistance, which occurs in the majority of patients during treatment. Most of the resistance mechanisms lead to a reactivation of the MAP kinase signaling pathway [50, 51].

Chemotherapy plays a subordinate role in the treatment of melanoma brain metastases. Before the approval of ipilimumab, prior to 2011, treatment with dacarbazine was considered the standard of care for patients with inoperable melanoma metastases [52]. Alternatively, temozolomide or fotemustin was used without leading to a significant prolongation of overall survival [53].

Generally, SRS is widely accepted in patients with 1–3, maximal 4 lesions. There is increasing evidence that even in multiple lesions, SRS can be highly effective. Considering the benefit of high local doses for radioresistant melanoma lesions, multiple SRS may be more effective compared to WBRT in those patients. Only recently, Yamamoto et al. [54] reported excellent outcomes of radiosurgery, even in patients with ten or more lesions. Further analyses have shown that outcome in patients with 5–10 lesions is non-inferior to patients with 2–4 lesions [48, 55]. Importantly, the addition of novel drugs such as TK-Is or other immunomodulators can be applied safely in the context of SRS without enhanced toxicity [56]. Here for SRS can be seen as a bridge to systemic treatment and should be considered for palliative therapy for patients with unresectable or metastatic melanoma. After an international meta-analysis of Lehrer et al. in 2018, 17 studies across 15 institutes in 3 countries have been analyzed and shown that concurrent administration of immune checkpoint inhibitors and SRS does not appear to be associated with untoward rates of radionecrosis with a possible survival advantage observed. Additionally, enhanced regional brain control within the brain with excellent rates of 1-year LC may be associated with concurrent therapy [57, 58]. This argues for non-invasive treatment of brain metastases even in advanced and extra-cranially spread disease to prevent progression and subsequent neurological deterioration, while treating the underlying disease effectively.

In conclusion, despite the still poor prognosis for brain metastasis in malignant melanoma, SRS is an effective treatment method. The BRAF biomarker and prognostic scores, such as the melanoma molGPA, could be used as tools in treatment decision making. The present survival outcomes support the use of disease-specific melanoma molGPA as reliable prognostic score. Immunotherapy is continuously revolutionizing outcome in patients with melanoma, making noninvasive and highly effective local treatments for brain metastases more relevant, even if the benefits and risks of the combination of radiotherapy with BRAF-targeted agents are also yet to be established. However, all patients with melanoma brain metastases should be discussed in a multidisciplinary team to make the best recommendations regarding patients’ treatment and care. Key research opportunities for future clinical trials are SRS in combination with BRAF pathway inhibitors and systemic therapies.

## Abbreviations

BRAF: Serine/threonine protein kinase encoded on chromosome 7q34, that activates the MAP kinase/ERK-signaling pathway
CA: Carcinoma
CT: Contrast-enhanced computed tomography
CTCAE: Common toxicity criteria
CTLA-4: Cytotoxic T-lymphocyte-associated protein 4
D_max_: Maximum dose to the PTV
DBC: Distant brain control
dsGPA: Disease-specific GPA
EFS: Event-free survival
FSRT: Fractionated stereotactic radiotherapy
GKR: Gamma knife radiosurgery
GPA: Graded prognostic assessment
IGRT: Image-guided radiotherapy
IQR: Interquartile range
KPS: Karnofsky performance score
LC: Local control
MM: Malignant melanoma
MVA: Multivariate analysis
NR: Not reported
OS: Overall survival
PD-1: Programmed cell death protein 1
PTV: Planning target volume
RCC: Renal cell cancer
RPA: Recursive partitioning analysis
RT: Radiotherapy
RTOG: Radiation therapy oncology group
SRS: Stereotactic radiosurgery
UVA: Univariate analysis
WBRT: Whole-brain radiotherapy
